# Physiology

**Published:** 1857-02

**Authors:** 


### PHYSIOLOGY.

*Lectures on Comparative Physiology*, by M. FLOURENS. Translated for Charleston Medical Journal and Review, by F. PEYRE PORCHER, M. D., Charleston, S. C.

SUMMARY.—*Paleontology—The* DEAD *and the* LIVING *periods in the History of the Earth—Ideas of Descartes and Leibnitz upon the Primitive Incandescence of the Globe.*

We have now reached the study of the fourth great question of Positive Ontology, to wit: the destruction of living beings in the different ages of the globe. After *Neontology*, we proceed to investigate *Paleontology*.

We know the present state of the animal population, which is distributed upon the face of the earth, according to the law of climates. The condition of things of to-day, has it always existed? No; the actual species have been preceded by other species, otherwise distributed upon the globe, and which numerous revolutions have successively destroyed. The same revolutions, in upsetting the surface of the earth, have accumulated ruins which form the soil upon which we now live—soil still scarcely settled; the tremblings of the earth, volcanoes which explode at intervals, are the weakened echoes of the grand commotion of other times.

The history of the globe comprehends two periods: 1st, that when life had not yet appeared—I call it the *dead* period; 2nd, that where life manifested itself—I call that the *living* period.

I will follow, in the examinations, not the order of time, but the order of our discoveries.

The first fact which has revealed to us a past differing from the actual arrangement, is the discovery of marine shells upon the dry earth. However little we penetrate the soil, they are everywhere to be found, even at great distances from the sea, and upon very considerable elevations.

The sea, at a certain epoch, actually covered the now dry earth; and, in retiring, it has left its shells deprived of their ancient inmates. The layers of earth which contain these marine shells are themselves other witnesses of the sea's sojourn. It is, indeed, the work of the waters; these are the sediments of those waters which formed them: thus we see them always disposed in horizontal lines.

Another essential circumstance: these horizontal layers having terminated at the foot of a mountain, there we find other layers more or less vertical. In other words: in the beginning, these oblique or vertical strata have been disposed horizontally; a cause which I will explain further on, has righted them up. They plunge under the first, where we found the layer of shells; and they themselves contain shells, but of species and genera very different.

The waters, then, have rested upon the face of the earth at different epochs.

This is not all. In searching more in advance, we reach strata which furnish the remains of mammifers; more profoundly still those of reptiles; then the remains of fish, and other shells still. The layers of marine animals alternate with the layers of terrestrial animals.

We are then warranted in concluding from all this that at different intervals the sea has successively covered the earth, and successively left it. It is easy, after the evidences furnished by observation, to conceive of what has been accomplished in these ante-historic epochs. The waters, in violently displacing themselves, on one side, left dry a marine population, whilst on the other, they submerged a terrestrial one. Frightful destructions, to which succeed others not less frightful: the sea, resuming her ancient bed, found there terrestrial animals that it destroyed in their turn; whilst that, behind it, numerous marine animals perished upon a soil given back once more to terrestrial life.

Such has been the *living* period.

All these facts are deduced rigorously from observation. Nothing in the preceding recitation has depended upon hypothesis. In fine, if we search to a still profounder depth, arriving at primitive strata, to that which constitutes the *ground-work* of the globe, we find no more animal remains. There was then an epoch when life did not exist upon the globe; and that is the *dead* period. This phase in the history of the globe, offers phenomena of a very different kind.

During the *living* period, water was the great agent which manifested itself. Its displacement caused immense destruction of living beings; the water produced the successive layers of sediment: it is that which has fashioned, so to speak, the globe as to its external envelope. During the *dead* period, the agent which showed itself was fire. Every thing bears proof, we will see, that in the beginning the globe was incandescent—liquified by fire throughout its mass.

Fire and water, these are the two forces which have acted in the infancy of the earth. Every effort, every object, even of Geology, is to separate, to day, in the contexture of the globe, that which was the effect of fire, from that which has been the effect of water.

Primitively, the globe was incandescent. This great idea, if, since a quarter of a century, we had not followed the progress of the science, would be enough to astonish us, and profoundly. Now we are familiarized with it.

The first to conceive so bold an idea, is Descartes. But with him this conception was not derived in any manner from the direct examination of the natural phenomena: it is a simple speculative application of certain physical laws which he had imagined: it bound itself with his celebrated system of *vortices*.

According to Descartes, all matter is composed of particles of three kinds: 1st, the finest for the *first element;* 2d, the globular particles, which are those bodies rounded by friction, form the *second element;* 3d, the largest pieces, and which preserve more angles, constitute the *third element*.

These elements, of which all space is full, move in *vortices*, the one around a centre, the others around another; each one at the same time, and at each instant, is drawn on by a centrifugal force.

The larger the matter is, the more it diverges from the centre. On the contrary, the finest powder (first element) has ranged itself at the centre and constitutes a *sun*. Each vortex has its collection of fine powder, its sun—all these *suns*—centres of as many vortices, that we call the *stars*.

The globular matter (second element) being composed of unequal globules, the largest diverge nearest the extremities of the vortex, the smallest maintain themselves nearest the sun; the fine dust which composes the sun communicates its agitation to the neighboring globules, and it is in that that *light* consists.

Lastly, in continuing these hypotheses, Descartes came to imagine little vortices of matter which rolled with the great. Each of these little vortices contained also globular matter, and, at the centre, an aggregation of fine dust which, in the beginning, formed a little *sun*. But as it contained, besides, heavy bodies, the brightness (bursting) of broken angles (third element,) these particles, collected together in thick clusters, gained the extremities of the little vortex, by the superiority of their centrifugal force; they obscured it; crusted it over little by little; and of these crusts thickened over all its surface, is formed an opaque body, a *planet*, a habitable *earth*. Thus the *earth is an encrusted sun.*

These ideas of Descartes are, as you see, purely abstract ideas. The great Philosopher of the XVIIth century, allied his ideas to fact; a method which has merit in Metaphysics, but which is not acceptable in Physics, nor in Natural History. In these Sciences, the power of ideas is essentially subordinate to the power of facts.*

Leibnitz has arrived at the same conception as Descartes, but by a different route, by observation.

The matters, melted and calcined, which are found within the entrails of the earth, had given to Leibnitz, the idea of a general conflagration. In his treatise entitled Protogœa,† Leibnitz said that the earth and other planets were, in the beginning, luminous stars by themselves. After having burned a long time, they became extinguished for want of combustible matter, and became opaque bodies. The fire has produced, by the melting of the matter, a vitrified crust. The base of all the matter which composes the earth is glossy : *facile intelligis vitrum esse velut terræ basin.*

If the presence of melted matter in the bosom of the earth had revealed to Leibnitz the primitive incandescence of the earth, another observation, that of the dispersion of fossil shells over the whole surface of continents, had given him the idea of a general submersion. When the crust of the earth was cooled, he tells us, the humid particles, which were raised in the form of vapor, fell back again, and enveloping the whole globe, constituted the seas.

Thus, Leibnitz had drawn from the observation of these two great facts, the conflagration and the submersion of the globe.

These ideas of the German thinker made no sensation at the time. The century was not prepared to receive them. The *Protogœa*, written in Latin, did not leave the bookshelves of the learned. It required, for the triumph of the ideas of Leibnitz, that Buffon should take them up in the second half of the XVIIIth century and give to them a new power, that of eloquence.

1. Obliteration of the Portal Vein and the Secretion of Bile.—At the meeting of the French Academy of Sciences, Sept. 1, 1856, M. Oré presented a paper, detailing a series of experiments, made with a view to ascertain the influence of obliteration of the portal vein, upon the secretion of bile. The experiments were undertaken at the instance of M. Gintrac, of Bordeaux, who had observed, in his practice, a case which seemed to contradict the ordinarily received physiological doctrine, which teaches that the portal vein furnishes the liver with the materials for the secretion of bile.

---

* The system of *vortices* was, in the XVIIth century, a subject of serious controversies. In the XVIIIth, Voltaire was not the last to laugh at it; we read in the *Dialogues* d'Evhemere : " *Evehemere :* Cardestes (Descartes) has supposed that our nest was at first an encrusted sun. *Callicrate :* A sun crusted over! you laugh. *Evehemere :* It is this Cardestes doubtless who jested when he said that we had been once a sun composed of matter subtle and matter globular ; but that, our matter being thickened, we have lost our brilliancy and our force : we have fallen from a vortex of which we were the centres and the masters, into the vortex of the sun of to-day : we are all covered with matter branching and channelled ; in fact, of the star that we were, we have become a moon, having around us, by favor, another little moon to console us for our disgrace."

Now the system of *vortices* is forgotten.

Descartes is not less the immortal author of the *Discours de la Methode.*

† This Treatise appeared in 1683, in the *Actes de Leipzig.*

The following are the author's conclusions :

"1. The secretion of bile having continued, after the partial and the complete obliteration of the portal trunk, I have concluded that it is not the blood of this vein which supplies the liver with material for its secretion. The gland is therefore dependent for its secretion upon the blood of the hepatic artery. The biliary secretion, like all others, is derived from the circulation of arterial blood. I have elsewhere established, that obliteration of the hepatic artery is not the proper means of testing the question, and cannot be considered, therefore, as weakening the conclusions which I am about to state.

"2. The secretion of sugar, by the liver, not having been altered by obliteration of the portal vein, is it not evident, as proved by M. Claude Bernard, that the production of saccharine matter is a proper secretion of the liver, and entirely independent of alimentation ?

"3. The albuminous and glycogenous matters resulting from the digestion of feculent and albuminoid substances, being prevented from passing through the liver, are nevertheless not lost to the system, but enter the inferior cava vein by an anastomotic branch from the superior mesenteric vein.

"4. Finally, and it is with great hesitation that I put forth the statement that arterial blood does not perform so important a part in the formation of sugar in the liver as in the secretion of bile."

After the reading of this paper, M. Andral stated, that in his medical practice, he had observed a fact which fully substantiated the conclusions cf M. Oré. A patient in whom there was reason to suspect, in consequence of external signs, an obliteration of the portal vein (a suspicion which was subsequently confirmed by an autopsical examination,) not only did not present symptoms indicative of a suspension of biliary secretion, but yet gave evidence of a persistence of the glycogenous function by the occurrence of a diabetes.—*Revue Médicale, Oct* 15, 1856. [Can it be possible that the distinguished medical savans of the French Academy, are ignorant of the fact, established by Kiernan and others, that the capillary terminations of the hepatic artery end in venous capillaries which open into the adjacent subdivisions of the portal vein, and are not, therefore, the radicles of the hepatic veins ? A knowledge of this circumstance would, it seems to us, have furnished sufficient explanation of the continuance of the biliary secretion after obliteration of the portal trunk. But even if this were not the case, another fatal objection to the conclusions of M. M. Oré and Andral is to be found in the fact that, when the portal vein is tied, its blood of course enters the general circulation, and in this way no small portion of it reaches the liver by the hepatic artery.—Ed.]

2. The Temperature of the Blood lowered by passing through the Lungs.—At the sitting of the same Academy, on the 15th of September, 1856, M. Claude Bernard read the continuatiou of his "Experimental Researches upon Animal Temperature," the second part of which relates to the modifications of temperature which the blood undergoes in traversing the respiratory organs. These experiments were made upon living animals, and M. Bernard thinks himself justified in coming to the following conclusions :

"1. That the circulation of the blood through the lungs causes a depression of the temperature of that fluid.

"2 That the lungs cannot therefore be considered a focus of animal heat.

"3. That the conversion of venous into arterial blood in the living animal, is not coincident with an augmentation in the temperature of that fluid, but, on the contrary, with a depression of its temperature."

In his next communication, M. Bernard proposes to examine into the modifications of temperature which the blood experiences in circulating through the genito-urinary apparatus.—*Ibid.*

[At a previous meeting of the Academy, M. Bernard, in a report of his experiments upon the modification of the temperature of the blood in its passage through the liver, established the fact that the blood is actually much warmer as it comes from that organ, than it is previous to its entrance.]

3. Coagulation of the Blood.—In a recent letter to the London Lancet, Dr. Fred. W. Headland proposes a theory in regard to the coagulation of the blood, opposed to that advocated by Dr. B. W. Richardson, in his prize essay.

Sir :—When, considering the subject of the chemical history of diabetes, in the year 1853, I was led, amongst other things, to conceive a certain theoretical explanation of the spontaneous coagulation of the blood. Having just now read over and reconsidered my notes taken at that time, I must say, that I still prefer my theory to the one recently promulgated by Dr. B. W. Richardson, which it resembles in some points.

The statement, which I append below, is copied nearly verbatum from the notes referred to, but I may premise that the part of the idea which applies to *vegetable* physiology, is the only part to which I have given any publicity , as at the time when it occurred to me I considered the whole matter of little moment.

The albuminous or nitrogenous matter found in the young growing cells of *a plant*, is in an insoluble condition. But on its passage in the fluids to those cells it must have been in some manner held dissolved in water. So, when the yonng cells get old and woody, they no longer contain this albuminous matter. On leaving them for the younger parts, it must again have assumed the soluble state. It seems to me that this phenomenon may be accounted for by supposing the albumen to be dissolved by an *alkaline* condition of the fluids, and deposited in some manner by the development of an *acid* at the particular point required.

The same may occur with the fibrine of animal blood, more prone to to such coagulation than albumen. In the tissues nourished by that blood it occurs in an insoluble form. But in the venous blood which carries away the effete products of those tissues, the fibrine is once more found in a state of solution. We are tempted to explain these facts in the same way as above: to suppose the fibrine held in solution by the presence of an alkali, and deposited in the insoluble form by the development of an acid. And, *in fact*, the blood is known to be feebly alkaline (from the presence of the carbonate or basic phosphate of soda,) and the juice of muscle was found by Liebig to have an acid reaction.

But how is it, that when the blood is withdrawn from the living body, its fibrine quickly assumes the solid or insoluble state—*i. e.* coagulates ? By parity of reasoning, I suppose that this takes place again, by the development of an acid, to such an extent as simply to neutralize alkalinity, not so as to precipitate the albumen; this acid I believe to be *lactic acid.* It has been lately shown that glucose is always present in the living blood; and further proved, as I believe, that this glucose is continually changing into lactic acid, which, as fast as it forms, is bound or combined with oxygen to support the animal heat. When the blood is isolated, this vital process of combustion goes on no longer, lactic acid accumulates, the alkalinity of the natural blood is neutralized, and the fibrine coagulates. A tendency to nitrogenous decomposition may hasten this process, by supplying a ferment to act upon the glucose. Blood drawn in fevers coagulates quickly. Diabetic urine, allowed to stand for a time, may lose its sugar, and contain lactic acid. Extensive coagulation of the blood in the living body may be caused by injecting a putrifying liquid into the veins.

Another time I propose to comment at length, upon the theory of Dr. Richardson, and to state the motives which induce me to dissent from it. I am also planning a series of experiments, in order to put to a satisfactory test, the truth or falsity of the hypothesis sketched above.—*The Lancet.*

